# Vitamin D and hippocampal development-the story so far

**DOI:** 10.3389/fnmol.2015.00058

**Published:** 2015-10-07

**Authors:** Anne L. Lardner

**Affiliations:** Department of Biochemistry, St Vincent’s University HospitalDublin, Ireland

**Keywords:** vitamin D, hippocampus, neurogenesis, differentiation, proliferation, LTP, depletion, repletion

## Abstract

Epidemiological studies suggest that vitamin D insufficiency may be prevalent in young as well as older populations. The pleiotropic effects of vitamin D are now beyond dispute and a growing number of studies provide accumulating evidence of a role for vitamin D in brain development and function. A number of studies to date have investigated the effects of early-life vitamin D deprivation on adult hippocampus in animals and humans, and there is a growing body of evidence to suggest a role for this hormone in the development of selected hippocampal functions such as latent inhibition and hole board habituation in rats. There are few studies to date of vitamin D deprivation or supplementation on early hippocampal development *in vivo*. However, a small number of studies, mostly *in vitro*, point to a role for vitamin D in differentiation and development of hippocampal neurons. There is also limited evidence that supplementation with vitamin D following a period of deprivation is capable of restoring cellular activity and later function. Further avenues of future research are outlined including animal studies on the effects of vitamin D deprivation and inadequacy on early hippocampal biochemistry and function, e.g., measurement of BDNF levels, GABAergic activity, long-term potentiation (LTP) and spatial navigation. It also remains to be established if there are critical developmental windows during which vitamin D is required. In light of the importance of the hippocampus in LTP and spatial learning, further investigations on the early effects of vitamin D deprivation on hippocampal development are warranted.

## Introduction

Until a little over 30 years ago, the role of hormonal vitamin D was classically associated with calcium homeostasis, bone formation and maintenance, its up-regulation by parathyroid hormone being known for many years. However in recent years, myriad other functions and roles for vitamin D have been gradually postulated and verified, and its pleiotropic effects are now beyond dispute (Kalueff and Tuohimaa, 2007; McCann and Ames, 2008; Lai and Fang, 2013; Schlogl and Holick, 2014). Some examples of its diverse range of effects include immune-modulatory and pro-differentiation activity, cellular regulation and apoptosis, anti-inflammatory and antimicrobial activity, insulin secretion, interaction with the renin-angiotensin system, neuroprotection*, inter alia*. (See for examples, DeLuca and Cantorna, 2001; Garcion et al., 2002; Holick, 2003; Lin and White, 2004; Nagpal et al., 2005; Norman, 2006). Vitamin D is now known to be synthesized in many cells such as skin, lymph nodes, colon, adrenal medulla and pancreas (see for example, Zehnder et al., 2001; Lai and Fang, 2013) and it regulates approximately 3% of the human genes via its endocrine effects (Bouillon et al., 2008).

Among the general population, pregnant women are considered to be at risk of vitamin D deficiency (VD). The increased demands of the developing fetus combined with a possible decrease in environmental exposure to sunlight, may result in diminished circulating vitamin D (Hillman and Haddad, 1976; Markestad et al., 1983). One large US study reported that 12% of women aged 25–29 had low serum 25-hydroxyvitamin D levels (Looker and Gunter, 1998) while another study of young Canadian women reported even higher prevalences (Vieth et al., 2001). A high prevalence of VD has also been reported in infants, children and adolescents from various countries (Huh and Gordon, 2008). In addition, women who wear concealed clothes are also at risk and in one study, hypovitaminosis was observed in 82.5% of such a cohort (Belaid et al., 2008). The question of reduced vitamin D concentrations on consequent neonatal, infant and child development in relation to the hippocampus is therefore highly pertinent.

## Vitamin D and the CNS

The earliest evidence for binding of Vitamin D3 within the nervous system was first obtained over 35 years ago by the seminal autoradiographic studies of Stumpf et al. (1979, 1980), who reported its accumulation in the posterior pituitary, forebrain, hindbrain and spinal cord. However an earlier study by Clemens et al had reported that antibody to the Vitamin D receptor (VDR) within the dorsal hippocampus was strikingly similar to the D3 receptor (Clemens et al., 1985). Further autoradiographic studies by Stumpf and O’Brien (1987) demonstrated the positive presence of nuclear Vitamin D staining in many other brain regions including the ventral hippocampus. On the basis of the extensive presence of vitamin D within the brain, they proposed that calcitriol be renamed solitriol, the sunlight activated steroid hormone, to reflect its wider physiological remit. Since then the investigation of a neural role for vitamin D has gained significant traction and a plethora of animal studies have shed light both on its presence and its biological activity within the CNS independent of transport via the blood-brain barrier. For example, a number of studies have revealed the expression of VDR in specific brain regions including the thalamus, cerebellum, amygdala, cingulated gyri, temporal lobe, cerebral cortex, and hippocampus (Clemens et al., 1988; Prüfer et al., 1999; Langub et al., 2001; Zehnder et al., 2001; Garcion et al., 2002; Eyles et al., 2003; McGrath et al., 2004). Biosynthetic and degradative enzymes for the vitamin were also reported in glial and neuronal cells *in vitro* (Clemens et al., 1988; Neveu et al., 1994a,b; Baas et al., 2000). An important study by Eyles et al established for the first time that the VDR and α-1 hydoxlyase enzyme co-localized in specific areas of the brain (Eyles et al., 2005). This was followed by several functional studies on physiological and cellular effects of vitamin D on various aspects of brain cellular function (for examples see Ko et al., 2004; Lin et al., 2005; Taniura et al., 2006).

A growing body of evidence also points to a role for vitamin D in the development of the CNS. Vitamin D3 receptors have been located in the CNS of the rat embryo (Veenstra et al., 1998). In addition, transient deprivation of vitamin D early in life leads to changes in the new-born rat brain which may persist into adulthood. The offspring of vitamin D deficient rats have larger lateral ventricles (Eyles et al., 2003; Féron et al., 2005), a thinner cortex (Eyles et al., 2003) diminished levels of nerve growth factor (NGF; Eyles et al., 2003; Féron et al., 2005), and reduced expression of a number of genes involved in neuronal structure and neurotransmission (Féron et al., 2005). These changes do not persist if young animals are fed a vitamin D replete diet at birth but persist if they remain on a vitamin D deficient diet for the first 3 weeks of life (Féron et al., 2005). VD has also been shown to interfere with cortical development in developing rat brains by inducing fewer apoptotic cells at birth and more mitotic cells overall (Ko et al., 2004). Targeted gene arrays specific for apoptosis and cell cycle genes confirm a pattern of transcription deregulation in the deplete group consistent with the known properties of vitamin D (Ko et al., 2004). The latter study suggests that the known pro-apoptotic and pro-differentiating properties of Vitamin D may also play a role in brain development. In addition, vitamin D has been demonstrated to increase levels of NGF and neurotrophins NT-3 and NT-4 in cultured glial cells, astrocytes and oligodendrocytes (Neveu et al., 1994a,b; Baas et al., 2000). Neurotrophins (NT) play an important role in the survival of developing neurons and in the proliferation and differentiation of neural progenitor cells (Davies, 2004). Its overall contribution to healthy brain development renders also likely a role for vitamin D in the developing hippocampus. A recent paper investigated the effects of calcitriol on neural stem cell differentiation from cultured mouse neurospheres, and reported concentration-dependent increases in the numbers of cells containing the intracellular neural marker NeuN, and the oligodendrocyte marker GalC, and the astrocyte marker GFAP. They also reported increased VDR expression within the neural stem cells (Shirazi et al., 2015).

## Studies on the Hippocampus

Functional VDRs were first reported in the dentate gyrus (DG), pyramidal and granule layers, glial cells and in subfields CA1–3 of the rat hippocampus, which were capable of specifically binding DNA response elements to osteopontin (Langub et al., 2001). Following the discovery of VDR within the hippocampus, a number of studies have investigated the effects of pre or perinatal VD or supplementation on aspects of hippocampal structure or function in rodents. Several studies have also been carried out on cultured hippocampal cells *in vitro*. Although many of the *in vivo* studies have been conducted on brains of adult rodents, changes in structure or function in adult brains provide circumstantial evidence for impaired development, as reported deficits represent the outcomes of earlier anatomical or physiological abnormalities extending into adulthood. Therefore they are germane to an overall understanding of the role of vitamin D in hippocampal development.

## Behavioral Studies

Several studies have investigated the effects of prenatal VD deficiency in rodents on cognitive and behavioral functions which directly or indirectly involve the hippocampus. In one animal model, offspring of female rats who had been subjected to a VD deficient diet from 6 weeks prior to mating until the birth of the litter were evaluated for behavioral changes at various time points following repletion of Vitamin D at birth. Significant impairment of both latent inhibition and hole-board habituation in vitamin-deprived rats was reported (Becker et al., 2005). The former is considered to be a measure of the ability to learn to ignore irrelevant stimuli. The latter is a form of non-associative learning in which there is a progressive diminution with repetition of a specific stimulus and also represents the ability to ignore irrelevant stimuli. Both functions are thought to constitute a central feature of schizophrenia (Becker et al., 2005) and involve the hippocampus (Oades and Isaacson, 1978; Weiner, 1990). However there was no impairment of spatial learning within a radial maze, nor on two-way active avoidance learning. Vitamin depleted rats also demonstrated superior ability to maintain previously learned rules in a brightness discrimination task. The authors concluded that exposure to low prenatal vitamin D has a selective impact on certain aspects of memory only, with disruption of latent inhibition, but no effect on memory acquisition and memory retrieval. The apparently superior ability of VD rats to maintain previously learned rules in a brightness discrimination task is an intriguing finding which needs to be replicated. However this link at present remains speculative, albeit tenable. Apropos its functional significance, it is tempting to speculate that it may constitute a compensatory physiological mechanism in the absence of sufficient sunlight. A later study by the same group investigated synaptic plasticity and long-term potentiation (LTP) in the DG of the hippocampus (Grecksch et al., 2009). Surprisingly, VD induced an enhancement of LTP using weak or strong titanic stimulation which persisted for more than 24 h. The authors concluded that memory acquisition and retrieval was unaffected and only latent inhibition disrupted. They also noted that the finding of enhanced LTP was in good agreement with the improved memory associated with the brightness discrimination task of their earlier study. Harms et al investigated the effect of prenatal VD on different neurological behaviors in two strains of 10 week old mice, and noted increased frequency of head-dipping in the hole board test in both strains, indicative of increased exploration and hippocampal involvement (Harms et al., 2012). The clinical significance of the latter has not been evaluated to date. In a separate study by the same group, one strain of mouse displayed spontaneous hyperlocomotion, pointing to intraspecies as well as inter-species differences in responses to developmental VD (Harms et al., 2008).

In light of the major role of the hippocampus in long-term memory formation, the apparent lack of effect of VD on spatial learning and memory formation and retrieval is an important finding which needs to be verified across species. An investigation of BDNF levels in such studies would also be useful, given its importance in spatial learning and hippocampal plasticity (Kang and Schuman, 1995a, b; Leal et al., 2015). It must be borne in mind also, that rats in the cited studies received some vitamin D from their mothers from the time of birth until weaning, and it is possible that this could have resulted in a partial restoration of hippocampal structure and function.

The hippocampus constitutes part of the neural circuitry responsible for the acoustic startle reflex (Swerdlow et al., 2001). One study to date has investigated the effects of vitamin D deprivation over time on this reflex in rats (Burne et al., 2004). Several groups of rats were treated and compared e.g., no depletion, replete at birth, replete at weaning, depleted until 10 weeks of age, or depleted between 5 and 10 weeks of age, and they found that only the combined prenatal and chronic postnatal vitamin D deprivation, but not early life hypovitaminosis on its own, resulted in an impaired response. This suggests that early VD for a limited period does not automatically produce any long-term adverse effects on brain function.

## Neuroanatomical and Neurochemical Studies

Harms et al investigated the effect of prenatal VD on brain anatomy in two strains of male and female new-born mice and found a significantly reduced hippocampal volume in females but not males. However the phenotype did not extend into adulthood, suggesting that either normal postnatal development or the reintroduction of vitamin D at birth may have corrected the deficit (Harms et al., 2012). The finding of reduced hippocampal volume in females is noteworthy and in need of further replication. Its clinical significance if any, is presently unknown and can only be elucidated in the context of further specific behavioral studies comparing hippocampal function and activity in females and males. One other study on the offspring of VD mice has demonstrated smaller lateral ventricles at 30 weeks but normal hippocampal volume (Fernandes de Abreu et al., 2010). There are no studies to date on the effects of VD on hippocampal volume in rats or other species.

Two animal studies to date have investigated the effect of VD on hippocampal neurogenesis. The first study, utilising a 1-α hydroxylase knock-out mouse model investigated the hippocampus of 8 week old mice born of vitamin D-depleted dams following injection with labeled bromodeoxyuridine (bdU; Zhu et al., 2012). They found a 50% reduction in the number of what were referred to as newborn neurons within the subgranular zone of the DG, alongside an increase in the number of apoptotic cells, 7 and 28 days post-injection. Subsequent dietary supplementation with vitamin D_3_ was able to prevent the reduction in bdU-labeled DG cells. This was paralleled by a decrease in NGF levels within the brain, leading the authors to postulate on a direct link between both phenomena. The decrease in NGF levels is consistent with previous studies in relation to brain development (Eyles et al., 2003; Féron et al., 2005). However the finding of increased numbers of apoptotic cells in the hippocampus is in contrast to the findings of decreased numbers of apoptotic cells at birth in the developing cortex (Ko et al., 2004) and with the known overall effects of vitamin D on apoptosis. A second study used rats to investigate the effect of gestational VD on their 10 week old offspring and also reported reduced neuronal proliferation within the subgranular layer of the DG (Keilhoff et al., 2010).

It appears that the negative effects of early transient VD on the hippocampus can be counteracted, under certain conditions at least, by subsequent repletion *in vivo* (Eyles et al., 2003; Burne et al., 2004; Féron et al., 2005; Harms et al., 2012; Zhu et al., 2012). This suggests that VD under certain conditions, exerts only temporary rather than long-term deficits in structure and function. It remains to be established if this compensatory ability is limited to specific ontogenetic windows of development. More carefully differentiated investigations like this one on various aspects of hippocampal development and function are necessary.

Two studies have investigated the effect of a single treatment of 50 μg of vitamin D on neurotransmitter concentrations in offspring of rats, and found that within the hippocampus, levels of serotonin and 5H1AA, but not dopamine or HVA, were negatively affected by the single treatment, which the authors referred to as hormonal imprinting (Tekes et al., 2009a,b). This is an interesting finding which warrants further exploration over longer periods with repeated hormone treatments. Apart from elucidating valuable data on the direct effect of vitamin D on neurotransmitter levels within the hippocampus, such studies carried out on young animals could also shed valuable light on hippocampal development when combined with behavioral testing. A recent study investigated calcitriol supplementation for 6 weeks in rats and reported higher GABA levels in the hippocampus and cortex (Jiang et al., 2014).

## *In Vitro* Studies

Several groups have investigated vitamin D supplementation of up to 100 nM on developing cultured hippocampal neurons (Brann et al., 1999; Brown et al., 2003; Marini et al., 2010). Two of these reported arrested or reduced mitosis and cell division along with accelerated neurite outgrowth and increased NGF production (Brann et al., 1999; Brown et al., 2003). These findings are consistent with an earlier study demonstrating the upregulation of the anti-mitotic cyclin-dependent kinase regulators p. 21 and p. 27 in a related cell line (Rots et al., 1999). Marini’s group reported translocation of vitamin D from the cytoplasm to the nucleus and back to the cytoplasm, which caused a delay in cell proliferation and induction of cell differentiation (Marini et al., 2010). They expanded their study to include a simultaneous investigation of cell cycle activity during Vitamin D supplementation. They found reduced expression of two proteins involved with proliferation (PCNA and cyclin D1) between 12–15 h post incubation compared to controls. This coincided with a 70% decrease in thymidine incorporation into DNA and with the G1/S phase of the cell cycle. There was a concurrent 16-fold increase in levels of Bcl2, a protein marker of cell differentiation, and NF-L, one of the neurofilaments involved in maintenance and remodeling of the neuronal cytoskeleton. They also reported an increase in the development of dendrites and axons over 5 days of culture compared to controls. In a later study, the same group found that when serum is withdrawn from cultured hippocampal cells, treatment with 100 nM vitamin D fails to trigger differentiation. However, increasing the dose to 400 nM allowed the interaction of the vitamin with its receptor, resulting in differentiation (Bartoccini et al., 2011). This finding points to differential effects of varying concentrations of vitamin D on cellular hippocampal development and warrant further exploration. Although the concentration of hormone used to supplement *in vitro* is higher than that detected in standard rat chow, vitamin D levels in humans have been found in more than one study to be elevated in pregnancy (Seki et al., 1991; Ardawi et al., 1997). It is therefore possible that local gradients of the hormone occur in the developing embryonic tissues and organs *in vivo* which may mirror if not exceed those used experimentally.

Cui et al examined the effect of VD on neuroprogenitor formation in cultured neonatal rat brain cells (Cui et al., 2007). They found an increase in the number of neurospheres formed in culture from the subventricular zone (SVZ), which was unaffected by subsequent supplementation with vitamin D. The authors concluded that vitamin D can regulate cellular proliferation in the developing brain. It is noteworthy that exogenous vitamin D supplementation of an initially deprived medium failed to elicit any cellular response. This contrasts with the restoration of normal development following vitamin D repletion in whole animals (Féron et al., 2005), and highlights the potential disparity between *in vitro* and *in vivo* findings. It may reflect the lack of any pool of pre-existing progenitor cells in the *in vitro* studies compared with the *in vivo* situation. Interestingly, VDR positive staining was localized within the progenitor cells of control and deplete neonate brains, particularly within the SVZ, and was unaffected by vitamin depletion.

One *in vitro* study has investigated the interaction of glucocorticoids with vitamin D in isolated hippocampal cells (Obradovic et al., 2006). Pre-treatment with 1 μmol vitamin D for 24 h substantially reduced the degree of dexamethasone—induced apoptosis and induced the activation of the p42/p44 MAPK complex which is involved in cellular differentiation. Vitamin D also abrogated the inhibitory effects of dexamethasone on the MAPK complex. The antagonistic effects of vitamin D on glucocoticoid action was postulated to have potential significance in treatment of cognitive impairments and major depression, both of which are accompanied by high amounts of circulating corticosteroids (Holsboer, 2000).

## Future Directions and Conclusions

In light of the accumulating animal and *in vitro* evidence to date, it is likely that vitamin D plays a role in hippocampal development *in vivo*. However, the majority of animal studies to date have been carried on animals from several weeks old and upwards, and there are insufficient data on the effects of VD on newborn and adolescent animals. This constitutes a major lacuna in our overall understanding of the role of vitamin D in the early development of the hippocampus and is an avenue ripe for exploration. There is a need for similar studies as those carried out to date on vitamin deficient new-born animals and continuing at regular timepoints to adulthood, to elucidate clearly how VD impacts on development *in vivo* both anatomically and structurally. Further anatomical studies are particularly necessary on early hippocampal neurogenesis and neuronal differentiation *in vivo* to confirm the *in vitro* findings.

It is possible that there are temporal variations in the effects of vitamin D such that particular time points along the spectrum of hippocampal development are more sensitive to its absence or its supply, than are others. There is a need for detailed studies investigating the effects of varying degrees of VD over time on hippocampal structure and function, given that VD in human subjects is invariably partial rather than total.

The overall behavioral findings to date with rodents on the effects of early VD on hippocampal function are complex and subtle, pointing to specific deficits such as latent inhibition and hole board habituation, rather than global disruption of function. These findings are also in need of replication to eliminate the possibility that some of these observations are species-specific rather than global. The finding, for example, of increased hyper-locomotion in one specific mouse species but not another (Harms et al., 2008) highlights the potentially differing effects of VD even within a single species.

Hippocampal structure and function is altered in schizophrenia, and the neuro-developmental hypothesis of schizophrenia suggests that an interaction between genetic and environmental factors during critical windows of development negatively impacts on brain development. Low prenatal vitamin D has been postulated as an epigenetic risk factor for same (McGrath, 1999), and transient VD is considered a developmental model in schizophrenia research. This has led to an important volume of work on this topic, some of which has been cited in this review in the context of the hippocampus. Hippocampal hyperactivity in schizophrenia has been demonstrated via neuroimaging and GABAergic mechanisms have been implicated in this effect (Heckers and Konradi, 2014). The recent finding of elevated GABA levels within the hippocampus and cortex following calcitriol supplementation in adult rats is noteworthy in this regard (Jiang et al., 2014). The effect of VD on neuronal activity and on GABA levels within the developing hippocampus *in vivo* is also worth investigating.

Given the known adverse effects of glucocorticoid excess on early brain development and the ensuing propensity to psychiatric illness later in life, another worthwhile avenue of research is the exploration of combined glucocorticoid excess with VD over varying time periods pre and post natally on hippocampal development and function *in vivo*. In particular, their combined effect on neural plasticity and LTP within the hippocampus needs to be elucidated.

It also remains to be established whether or not prenatal or perinatal VD may result in subtle changes in the developing hippocampus which render it more susceptible to eventual development of neurodegenerative diseases such as Alzheimer’s and Parkinson’s. While a number of studies have looked at the effects of VD on cognitive function in ageing or elderly populations, none to date have attempted to ascertain whether VD in early life could predispose to cognitive dysfunction in adulthood. Such studies of VD in the developing hippocampus should also measure BDNF levels, which are known to decrease in hippocampal neurons of Alzheimers patients (Siegel and Chauhan, 2000).

Finally, there are no cross-sectional studies to date in young children on hippocampal function such as memory formation and retrieval, and spatial navigation as a function of their vitamin D intake. This would shed the most valuable light possible on the role of vitamin D in early hippocampal development.

## Conflict of Interest Statement

The author declares that the research was conducted in the absence of any commercial or financial relationships that could be construed as a potential conflict of interest.
